# Higher adherence to a Nordic diet is associated with reduced risk of mild depressive symptoms: a population-based analysis

**DOI:** 10.1007/s00394-026-04107-3

**Published:** 2026-10-07

**Authors:** Johanna Mylläri, Jyrki K. Virtanen, Niina E. Kaartinen, Tuija Jääskeläinen, Outi Nuutinen, Pilvikki Absetz, Satu Männistö, Anu Ruusunen, Timo Partonen

**Affiliations:** 1 Institute of Public Health and Clinical Nutrition, University of Eastern Finland, Kuopio, Finland; 2 Department of Public Health, Finnish Institute for Health and Welfare, Helsinki, Finland; 3 Faculty of Social Sciences, Tampere University, Tampere, Finland; 4 Mental Health and Wellbeing, Kuopio University Hospital, Wellbeing Services County of North Savo, Kuopio, Finland; 5 Deakin Institute for Mental and Physical Health and Clinical Translation (Deakin IMPACT), Food & Mood Centre, School of Medicine, Deakin University, Geelong, VIC Australia; 6 Department of Healthcare and Social Welfare, Finnish Institute for Health and Welfare, Helsinki, Finland

**Keywords:** Adults, Depression, Diet quality, Nordic Diet Index, Nordic diet, Population-based study

## Abstract

**Purpose:**

Although the Mediterranean diet has been widely studied in relation to depression, less is known about the associations between other diets, such as the Nordic Diet, and depressive symptoms and clinical depression. The aim was to examine whether adherence to a Nordic diet (assessed with the modified Nordic Diet Index, mNDI) is associated with depressive symptoms and clinical depression in the Finnish Health 2000–2011 survey.

**Methods:**

The cross-sectional data included 4697 adults, while the prospective data included 1788 participants for depressive symptoms and 1862 for depressive disorders. Depressive symptoms were assessed in 2000 using a composite variable derived from validated questionnaires, and in 2011 using the Beck Depression Inventory-13 (BDI-13). Clinical diagnoses were based on the Munich-Composite International Diagnostic Interview (M-CIDI) or self-report.

**Results:**

At baseline, 1746 participants (37.2%) reported elevated depressive symptoms. Higher mNDI (indicating better adherence) was associated with 25.0% lower multivariable-adjusted odds of depressive symptoms (Q4 vs. Q1; 95% CI 8.6–38.5%, p-trend = 0.013). In the prospective analyses, higher mNDI was associated with lower odds of elevated depressive symptoms (BDI-13 cut-off scores 1 and 5), but these associations attenuated after full adjustment. No association was observed at the BDI-13 cut-off 9 (all *p*-values ≥ 0.303). Adherence to a Nordic diet was not associated with clinical depression.

**Conclusion:**

Higher adherence to a Nordic diet was associated with fewer depressive symptoms and a reduced risk of elevated depressive symptoms, but not with clinical depression, possibly due to the limited number of cases diagnosed via M-CIDI or reported by participants (*n* = 82).

**Supplementary Information:**

The online version contains supplementary material available at https://doi.org/10.1007/s00394-026-04107-3.

## Introduction

Depressive disorder is a leading global health concern, adversely affecting life expectancy [1], physical and psychological well-being [2], and overall quality of life [3], while creating a significant economic burden [4]. Its high prevalence and the limitations of current treatment approaches highlight the need for effective prevention strategies [5]. Diet has emerged as a promising modifiable factor in the prevention of depressive symptoms, possibly through biological pathways such as modulation of the gut microbiome, inflammation, and oxidative stress [6]. Diet indices provide a practical way to study links between diet and depression because they capture overall eating patterns rather than single nutrients [7]. This approach aligns with the multifactorial biology of depression and offers a more realistic measure of habitual diet [6, 8].

Various systematic reviews and meta-analyses involving diverse populations have shown inverse relationships between diet quality and depressive symptoms [8, 9]. The strongest evidence supports the Mediterranean diet [8, 9]. Molero et al. [9] examined 21 trials and 92 prospective cohorts, totaling over 700,000 individuals, and found that higher adherence to a Mediterranean diet, measured by the Mediterranean Diet Score (MDS), was linked to a lower risk of depression. Similarly, Lassale et al. [8] conducted a meta-analysis of 20 longitudinal and 21 cross-sectional studies, discovering that greater adherence to the Mediterranean diet, assessed with four different diet scores, reduced depression risk by 33%. These scores reflect how closely people follow a Mediterranean dietary pattern, with higher scores indicating more frequent consumption of characteristic foods like vegetables, fruits, legumes, whole grains, fish, and olive oil, and less intake of less typical foods such as red and processed meats. Furthermore, a lower Dietary Inflammatory Index (DII) score was associated with a 24% decrease in depression risk. Additionally, Yu et al. [10] performed a meta-analysis of 11 cohort and 32 cross-sectional studies across Asia, North America, Europe, and Australia, reporting that higher DII scores correlate with increased depression risk through a nonlinear dose–response relationship.

Due to differences in food cultures and possible challenges of directly adopting Mediterranean diets in other parts of the world, there is a continuous need to study health-promoting diets based on different food cultures and food production premises. Nordic diet is similar to the Mediterranean diet in its emphasis on plant-based foods, moderate consumption of fish, eggs, and dairy, and restrictions on red meat and sweets. The Nordic diet favors the use of crop species adapted to northern climates, along with regionally sourced plant- and animal-based foods; rapeseed oil, whole grains like rye, oats and barley, berries like bilberries, lingonberries, and cloudberries, along with fruits like apples and pears, vegetables like cabbage and root vegetables, fatty fish such as salmon, and legumes like beans and peas.

Only a few studies have examined the link between the Nordic diet and depression [11–14]. A cross-sectional study among community-dwelling adults in Norway by Jacka et al. [11] found an inverse relationship between diet quality scores and depressive symptoms in both women and men. Another cross-sectional study of young, healthy women from Iran found no association between adherence to the Nordic diet and depressive symptoms [12]. In contrast, another study from Iran reported that higher adherence to the Nordic diet was associated with fewer depressive symptoms [13]. However, this was a case-control study, and the association was observed only in the case group of recovered COVID-19 patients. The most recent study among middle-aged or older Finnish men in Northern Savo, Finland, found that higher adherence to a Nordic diet, measured by a healthy Nordic diet score [15], was associated with a lower prevalence of depressive symptoms [14].

Due to limited research on the link between Nordic diet and the risk of depressive symptoms and clinical depression, we aimed to study whether adherence to the Nordic diet, assessed by the modified Nordic Diet Index (mNDI), is associated with the risk of depressive symptoms in the Finnish adult population. Additionally, we analyzed how mNDI predicts the development of depressive symptoms and a diagnosed depressive disorder over an 11-year follow-up.

## Materials and methods

### Study population

**Fig. 1:**
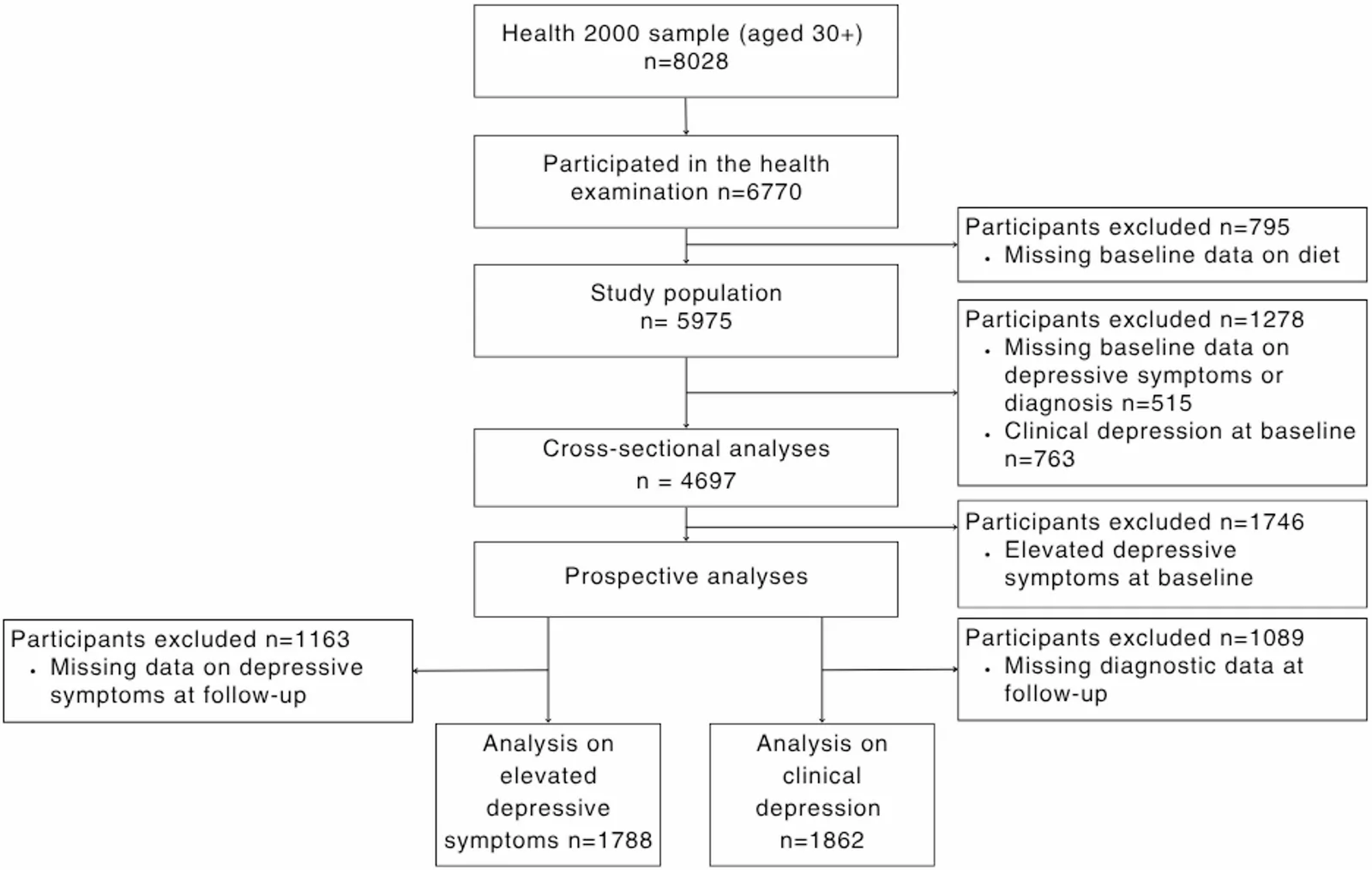
A flowchart showing the reasons for exclusion of participants from the analyses of the current study

The present study is based on a nationally representative sample of Finnish adults from the Health 2000 Survey, conducted in 2000–2001, and its follow-up, the Health 2011 Survey (H2011), conducted in 2011–2012. In 2000, a two-stage stratified cluster sample was drawn from the Finnish nationwide population register. All members of the H2000 sample who were still living in Finland in 2011 and had not declined participation in follow‑up studies were invited to the H2011 study. These studies used interviews, health examinations, and questionnaires [16, 17]. For this study, we included adults aged 30 and older living in mainland Finland who participated in the Health 2000 health examination and had reported their baseline data on diet, depressive symptoms, and clinical depression (Fig. 1).

### Dietary assessment

Food consumption was evaluated using a validated semi-quantitative Food Frequency Questionnaire (FFQ) [18, 19]. Participants reported their typical diet over the past 12 months. The FFQ included 125 common food items, mixed dishes, and alcoholic beverages. Participants indicated their food intake frequency on a nine-point scale: from *never or rarely* to *6 or more times daily.* Predefined portion sizes on the questionnaire helped participants to estimate serving sizes. The FFQ was administered to individuals participating in health examinations, with instructions provided. Participants completed the FFQ at home and returned it to the National Public Health Institute. Food consumption and nutrient intakes were calculated using the Finnish National Food Composition Database (FINELI^®^) and the FINESSI software [20]. Daily energy (kj) and alcohol (g) intakes were derived from the FFQ.

### The modified Nordic Diet Index

The specific components and scoring scheme of the mNDI were based on the Baltic Sea Diet Score (BSDS) [21, 22]. The mNDI was developed to assess adherence to a healthy dietary pattern based on Nordic dietary recommendations [21]. The mNDI included eight dietary components: fruits, vegetables, whole grains, low-fat milk, fish, red and processed meats, the ratio of polyunsaturated fatty acids (PUFA) to saturated fatty acids (SAFA) plus trans fats, and alcohol [21]. Components were scored using sex-specific intake quartiles, with scores ranging from 0 to 3, except for alcohol, which scored 0 or 1. Fruits, vegetables, whole grains, low-fat milk, fish, and the ratio of PUFAs to SAFAs + trans fats received higher scores for higher intakes (0 = lowest quartile; 3 = highest), whereas red and processed meats were scored inversely. Alcohol was scored 1 if daily intake did not exceed 20 g for men and 10 g for women; otherwise, it was scored 0. The total possible mNDI score ranged from 0 to 22. A higher score indicates a higher adherence to the healthy Nordic diet.

### Assessment of depressive symptoms and depressive disorders

Several measures were employed to evaluate depressive symptoms. The baseline questionnaire covered common symptoms, mood states, and seasonal variation in mood and behavior. In the Health 2000 study, depressive symptoms were assessed using specific questions from standardized scales, the Symptom Checklist 90 (SCL-90) [23, 24], the General Health Questionnaire (GHQ-12) [25], and the total score from a modified version of the Beck Depression Inventory [26, 27]. In the Health 2011 survey, the Beck Depression Inventory (BDI-13) [28] was used.

In both studies, the diagnosis of depressive disorder was obtained through the computer-assisted Munich-Composite International Diagnostic Interview (M-CIDI) [29], which was administered by specially trained nurses to participants who reported experiencing depressive symptoms within the past 12 months.

Additionally, in both studies, six items from the Seasonal Pattern Assessment Questionnaire (SPAQ) [30] asked about the degrees of seasonal variations in mood and behavior. The SPAQ is a widely used self-report tool that assesses the severity of seasonal changes in mood, appetite, weight, sleep duration, social activity, and energy levels, and screens for seasonal affective disorder (SAD). For these surveys, the original questionnaire was modified as follows: each item was a Likert-like scale scored as zero (no variation) to 3 (marked variation), yielding the total sum score (Global Seasonality Score, GSS) from 0 to 18. The higher the GSS, the higher the degree of seasonality. In addition, the item on the seasonal variations experienced as a problem was scored with a Likert-like scale of zero (no problem) to 4 (severe problem). For this analysis, a GSS equal to or higher than 11, plus a problem of at least a moderate degree, was defined to be equal to SAD in severity, whereas: (a) a GSS equal to or higher than 11, plus no problem at all or a problem of mild degree at its highest, or (b) a GSS of 9 to 10, plus a problem of at least a mild degree, was defined to be equal to subsyndromal SAD in severity. Otherwise, individuals had normal seasonality. This definition agrees with the Kasper–Rosenthal criteria [31], as it relies on similar wording for the items than the original SPAQ in classifying individuals into the three groups based on their scores.

### Other measures

Information on age (years) and sex (male/female) was derived from the sampling frame. Data on sociodemographic factors, including the highest level of education attained (basic/middle/high) and marital status (married/cohabiting, divorced/separated/widowed/single), as well as smoking status (regular smoker or non-smoker), were collected via a questionnaire. Body mass index (BMI; kg/m^2^) was calculated using either measured height (cm) and weight (kg) obtained during the health examination. Self-reported height and weight were used when measurements were unavailable.

The total leisure-time physical activity was evaluated based on two questions: (1) the frequency of vigorous physical exercise, asking, “How often do you exercise at least 30 minutes so that you sweat and get out of breath?” with response options including *“Daily*,*” “4–6 times per week*,*” “2–3 times per week*,*” “Once a week*,*” “2–3 times per month*,*” and “Few times per year or less frequently* (2) Commuting physical activity, assessed with the question, “How many minutes do you walk or cycle to and from work daily?” Response options were: *“I am not working or I work from home*,*” “Use public transport or car during commuting*,*” “Less than 15 minutes per day*,*” “15–29 minutes per day*,*” “30–59 minutes per day*,*” “1–2 hours per day*,*”* and “*Over 2 hours per day.”* Based on participants’ responses, leisure-time physical activity was categorized as ideal, adequate, uncertain, or inadequate.

### Statistical analyses

We used independent-samples t-tests and ANOVA for continuous variables and a Chi-Square test for categorical variables to examine differences in baseline characteristics across participants in different mNDI quartiles. To investigate both cross-sectional and longitudinal associations, we conducted concurrent and prospective analyses.

In the cross-sectional analysis, the association between baseline depressive symptoms and diet quality (mNDI) was examined using logistic regression, with depressive symptoms treated as a binary dependent variable. The mNDI was initially analyzed as a continuous variable and then as a categorical variable divided into quartiles. Linear associations were evaluated by modeling mNDI categories as a continuous ordinal variable to obtain a p‑for‑trend. Non‑linearity was tested by comparing linear and four‑level categorical logistic models using a likelihood ratio test (df = 2). *p* < 0.05 indicated non‑linearity. Participants were considered to have depressive symptoms if they reported feeling down or depressed during the past month (Question from SCL-90), feeling unhappy or depressed in the previous few weeks (Question from GHQ-12), and had a score ≥ 1 on the BDI questionnaire at baseline. Participants were classified as clinically depressed if they had either a diagnosis of major depressive disorder, dysthymia, or depressive disorder not otherwise specified as assessed with a diagnostic interview (M-CIDI) as conducted by study personnel, or the self-report of a diagnosis of or treatment for depressive disorder (ICD-10) by a physician within the past year.

In the prospective analysis, we used logistic regression to estimate odds ratios (ORs) for elevated depressive symptoms based on BDI-13 cut-offs of 1, 5, and 9, and for clinical depression risk, treating mNDI first as a continuous variable and second as quartiles. Analysis of more than one cut-off value for the BDI-13 scores allowed us to evaluate the association across levels of symptom severity.

Potential confounders identified from the literature include age, sex, education, marital status, smoking status, physical activity, BMI [32–38], and energy intake, since the latter was not part of the mNDI. Model 1 was adjusted for age, sex, and daily energy intake (kJ/d). Model 2 included Model 1 and socioeconomic factors; educational level, and marital status. Model 3 further included lifestyle behaviors; current smoking habits, leisure-time physical activity, and BMI. The models were based on the consideration that socioeconomic factors remain relatively stable structural determinants during adulthood and influence the context in which lifestyle behaviors and their changes occur. Age, daily energy intake, and BMI were treated as continuous variables, and educational level, marital status, physical activity, and smoking habits were treated as categorical variables. Missing data for BMI (*n* = 33), educational level (*n* = 27), marital status (*n* = 19), physical activity level (*n* = 125), and smoking habits (*n* = 31) were imputed using multiple imputation by chained equations (MICE) in SPSS, with 10 imputed datasets. Each dataset was analyzed separately, and results were pooled using Rubin’s rules to obtain final estimates [39, 40].

Individuals aged ≥ 30 years with complete baseline data on diet (administered in health examination), clinical diagnosis, and depressive symptoms were included in the cross-sectional analysis (Fig. 1). Participants with clinical depression at baseline (*n* = 763) were excluded to avoid confounding by pre-existing condition, leaving a sample of 4697 participants for the cross-sectional analysis (Fig. 1).

For prospective analysis of the risk of elevated depressive symptoms, participants with elevated depressive symptoms at baseline (*n* = 1746) and missing follow-up data on BDI-13 (*n* = 1163) were excluded, resulting in a final sample of 1788. For clinical depression, participants with missing diagnostic follow-up data were excluded, leaving 1862 participants for the analysis. The statistical significance of potential sex-specific interactions was assessed through stratified analysis and likelihood ratio tests using a multiplicative interaction term. The potential interaction between mNDI and BMI was assessed by including an interaction term in the logistic regression model. Sensitivity analyses were conducted by excluding all participants who were taking medication for a psychiatric or mental health condition at baseline. *P*-values below 0.05 were considered statistically significant. Data were analyzed using SPSS 27.0 for Windows (Armonk, NY: IBM Corp.).

## Results

### Baseline characteristics

Slightly more than half of the participants were women, and 54% were married (Table 1). One third had a basic level of education. About 20% were current smokers, and fewer than a third engaged in adequate or optimal leisure-time physical activity. The mean age was 49 years (SD 17.1), the average BMI was 26.5 kg/m^2^ (SD 7.0), and the average diet quality score was 11.4 (SD 3.6). Those with higher adherence to a Nordic diet (assessed with the mNDI) were more likely to be married, non-smokers, and more physically active in their leisure time (Table 1).

**Table 1:** Baseline characteristics according to the quartiles of the mNDI score

			mNDI quartiles (points)							
	All n = 5975		Q1 (≤ 8) n = 1271		Q2 (9–10) n = 1693		Q3 (11–13) n = 1702		Q4 (≥ 14) n = 1309	
	Mean	SD	Mean	SD	Mean	SD	Mean	SD	Mean	SD
Age (years)	52.8	15.1	50.1	15.5	51.6	15.4	53.6	14.9	56.1	13.9
BMI (kg/m^2^)^1^	26.9	4.7	26.6	4.9	26.7	4.4	27.2	4.6	27.3	4.7
Energy intake (kJ/d)	9571	3358	8225	2819	9063	2898	9987	3363	10,994	3724
Alcohol intake (g/d)	5.2	10.3	6.8	13.7	5.4	10.9	5.0	8.7	4.0	6.6
GSS^2^	5.02	3.05	4.76	3.13	4.95	3.0	5.15	3.08	5.19	3.01
	n	%	n	%	n	%	n	%	n	%
Sex (female)	3326	55.7	699	21.0	967	29.1	932	28.0	728	21.9
Marital status (married)^3^	3563	59.6	642	18.0	1007	28.3	1021	28.7	893	25.1
Education (basic)^4^	2320	38.8	507	21.9	648	27.9	659	28.4	506	21.8
Smoking (regular)^5^	1234	20.7	414	33.6	385	31.2	290	23.5	145	11.8
Leisure time physical activity (adequate or ideal)^6^	1864	31.2	309	16.6	461	24.7	535	28.7	559	30.0
SAD^7^	70	1.2	16	22.9	15	21.4	18	25.7	21	30.0

mNDI, modified Nordic Diet Index; BMI, body mass index; GSS, Global Seasonality Score; SAD, seasonal affective disorder

^1^missing n = 33

^2^missing n = 266

^3^missing n = 19

^4^missing n = 27

^5^missing n = 31

^6^missing n = 125

^7^missing n = 293

**Table 2:** Odds ratios for depressive symptoms, assessed based on responses to SCL-90, GHQ-12, and the total score of BDI, in quartiles of diet quality scores, N = 4697

	mNDI quartile					mNDI^b^	
	Q1 (n =974)	Q2 (n = 1320)	Q3 (n =1356)	Q4 (n =1047)	*P* for trend	OR (95% CI)	*p*-value
*Median diet quality score*	7	10	13	16			
*Depressive symptoms (%)*	22.2	28.1	29.6	20.0			
N of cases	388	491	517	350			
OR, Model 1^a^	1	0.877 (0.739–1.041)	0.897 (0.754–1.067)	0.713 (0.589–0.864)	0.002	0.970 (0.953–0.987)	<0.001
OR, Model 2	1	0.877 (0.738–1.042)	0.901 (0.757–1.073)	0.718 (0.592–0.871)	0.003	0.970 (0.953–0.988)	<0.001
OR, Model 3	1	0.889 (0.748–1.057)	0.925 (0.775–1.103)	0.750 (0.615–0.914)	0.013	0.976 (0.959–0.994)	0.008

SCL-90, Symptom Checklist-90; GHQ-12, General Health Questionnaire-12; BDI, Beck Depression Inventory; mNDI, modified Nordic Diet Index

^a^Values are odds ratios (95% confidence interval)

^b^Odds ratio (95% confidence interval) for each one-point increase in the mNDI

Model 1 adjusted for energy intake, age, and sex

Model 2 adjusted for Model 1 and education level and marital status

Model 3 adjusted for Model 2 and physical activity level, regular smoking, and BMI

### Prevalence of depressive symptoms and clinical depression at baseline

At baseline, 1746 participants (37%) reported experiencing depressive symptoms (assessed based on responses to SCL-90, GHQ-12, and the total score of BDI), while 1010 (14%) were classified as having clinical depression based on M-CIDI interviews or self-report. Among those without depressive symptoms, 135 individuals were still classified as having clinical depression through M-CIDI interviews or self-report. When evaluated by sex, 747 men (29.1%) and 999 women (33.9%) reported depressive symptoms (*p* for difference < 0.001) (Supplementary Material 1). In the stratified analyses, each one-unit increase in mNDI was associated with a 3.5% (95% CI, 1.1–5.8%) lower odds of depressive symptoms in women, whereas no association was observed among men (Model 3, Supplement). However, there was no interaction by sex (*p* = 0.207). Additionally, 70 participants were considered to have SAD, and 200 participants had subsyndromal SAD.

### Associations between mNDI and depressive symptoms

When mNDI was treated as a continuous variable, each one-point increase in the diet score was associated with a 3.0% (95% CI 1.3–4.7%) lower odds of depressive symptoms, adjusted for energy intake, age, and sex (Model 1) (Table 2). The associations remained unchanged after additional adjustments (Models 2 and 3). When mNDI was evaluated in quartiles, those in the highest quartile had 25% lower odds of depressive symptoms (95% CI 0.62–0.91, p for trend = 0.013, Model 3) compared to those in the lowest quartile.

### Risk for elevated depressive symptoms

During follow-up, elevated depressive symptoms, based on the BDI-13 cut-offs of 1, 5, and 9, were observed in 994 (55.6%), 160 (8.9%), and 46 (2.6%) participants, respectively. The events based on the BDI-13 cut-offs 1 and 5 were slightly more frequent in women than in men (Supplement). In the whole population, adherence to the Nordic diet, assessed with mNDI, was associated with a lower risk of elevated depressive symptoms at lower BDI‑13 thresholds (Table 3). At cut‑off 1, the association was statistically significant in Model 1 (OR 0.966; 95% CI 0.938–0.994, *p* = 0.018) and in Model 2 (OR 0.965 (95% CI 0.937–0.994; *p* = 0.049), but the association became weaker and non-significant in Model 3. At cut‑off 5, higher mNDI was linked to lower odds in Model 1 (OR 0.931; 95%CI 0.887–0.977; *p* = 0.004) and Model 2 (OR 0.944; 95%CI 0.898–0.992; *p* = 0.022), but not in Model 3. No statistically significant associations were observed at cut‑off 9 in any models. Similar results were observed when mNDI was analyzed in quartiles (Table 3); at cut‑off 1, statistically significant associations were observed in Model 1 (p for trend = 0.017) and Model 2 (*p* for trend = 0.018), and at cut‑off 5 in Model 1 (p for trend = 0.020). Again, no statistically significant associations were found at the BDI‑13 cut‑off of 9. There was a statistically significant interaction by sex at the BDI-13 cut-off of 9, although the associations were not statistically significant in either men or women (Supplementary Material 1). There was no interaction by sex at the BDI-13 cut-off of 5, nor were there significant associations in either sex. With the BDI-13 cutoff of 1, mNDI was associated with 4.6% (95% CI 0.4–8.5%) lower odds for depressive symptoms in women, whereas no association was observed in men. However, the interaction was not statistically significant (*p* = 0.159).

**Table 3:** Odds ratios for elevated depressive symptoms based on BDI-13 cut-off scores 1, 5, and 9, and mNDI scores treated as quartiles and as continuous, N=1788

	mNDI quartile					mNDI^b^	
	Q1 (n =350)	Q2 (n = 527)	Q3 (n = 270)	Q4 (n = 420)	*P* for trend	OR (95% CI)	*p*-value
*Median diet quality score*	7	10	13	16			
*BDI-13 CUT OFF 1*							
*Depressive symptoms (%)*	18.8	30.6	27.1	23.4			
N of cases	187	304	270	233			
OR^a^, Model 1	1	1.055 (0.797–1.397)	0.854 (0.639–1.141)	0.735 (0.538–1.004)	0.017	0.966 (0.938–0.994)	0.018
OR, Model 2	1	1.064 (0.803–1.411)	0.858 (0.641–1.148)	0.737 (0.537–1.010)	0.018	0.965 (0.937–0.994)	0.049
OR, Model 3	1	1.057 (0.795–1.404)	0.887 (0.660–1.192)	0.776 (0.561–1.074)	0.059	0.972 (0.943–1.001)	0.061
*BDI-13 CUT OFF 5*							
*Depressive symptoms (%)*	21.9	32.5	24.4	21.3			
N of cases	35	52	39	34			
OR, Model 1	1	0.875 (0.553–1.385)	0.649 (0.397–1.062)	0.579 (0.341–0.984)	0.020	0.931 (0.887–0.977)	0.004
OR, Model 2	1	0.939 (0.590–1.494)	0.715 (0.434–1.178)	0.671 (0.392–1.147)	0.077	0.944 (0.898–0.992)	0.022
OR, Model 3	1	0.967 (0.606–1.545)	0.779 (0.471–1.291)	0.816 (0.469–1.417)	0.321	0.962 (0.914–1.013)	0.138
*BDI-13 CUT OFF 9*							
*Depressive symptoms (%)*	23.9	32.6	19.6	23.9			
N of cases	11	15	9	11			
OR, Model 1	1	0.832 (0.374–1.850)	0.511 (0.205–1.271)	0.672 (0.271–1.662)	0.252	0.955 (0.876–1.042)	0.303
OR, Model 2	1	0.933 (0.415–2.099)	0.602 (0.239–1.517)	0.865 (0.346–2.164)	0.545	0.979 (0.897–1.069)	0.643
OR, Model 3	1	0.938 (0.417–2.113)	0.630 (0.249–1.594)	0.969 (0.378–2.481)	0.716	0.991 (0.905–1.085)	0.845

BDI-13, Beck Depression Inventory 13; mNDI, modified Nordic Diet Index

^a^Values are odds ratios (95% confidence interval)

^b^Odds ratio (95% confidence interval) for each one-point increase in the mNDI

Model 1 adjusted for energy intake, age, and sex

Model 2 adjusted for Model 1 and education level and marital status

Model 3 adjusted for Model 2 and physical activity level, regular smoking, and BMI

**Table 4:** Odds ratios for clinical depression, assessed by M-CIDI or self-report, in quartiles of mNDI scores during an 11-year follow-up, N = 1862

	mNDI quartile					mNDI^b^	
	Q1 (n =363)	Q2 (n = 549)	Q3 (n =512)	Q4 (n =438)	*P* for trend	OR (95% CI)	p value
*Median diet quality score*	7	10	13	16			
*Clinical depression* (%)	28.4	29.6	17.3	25.9			
N of cases	23	24	14	21			
OR, Model 1^a^	1	0.714 (0.393–1.296)	0.454 (0.226–0.912)	0.875 (0.449–1.703)	0.425	0.987 (0.922–1.056)	0.700
OR, Model 2	1	0.727 (0.399–1.323)	0.456 (0.226–0.919)	0.872 (0.445–1.709)	0.416	0.986 (0.921–1.056)	0.986
OR, Model 3	1	0.722 (0.395–1.320)	0.473 (0.233–0.962)	0.936 (0.464–1.887)	0.540	0.995 (0.926–1.068)	0.995

M-CIDI, Munich-Composite International Diagnostic Interview; mNDI, modified Nordic Diet Index

^a^Values are odds ratios (95% confidence interval)

^b^Odds ratio (95% confidence interval) for each one-point increase in the mNDI

Model 1 adjusted for energy intake, age, and sex

Model 2 adjusted for Model 1 and education level and marital status

Model 3 adjusted for Model 2 and physical activity level, regular smoking, and BMI

### Risk for clinical depression

In the whole population (*N* = 1862), there were 82 (4.4%) clinical depression events. Over an 11-year follow-up, there was a statistically significant difference (*p* = 0.003) in events between men (*n* = 28, 30.0%) and women (*n* = 54, 58.1%)(Supplement). When mNDI was analysed in quartiles, no statistically significant differences were found between the highest and lowest categories in any models (Table 4). There was a statistically significant 53% lower odds for clinical depression in the third mNDI quartile (95% CI 3.8–76.7%, Model 3), suggesting a non-linear association; however, the *p*-value for non-linearity did not reach statistical significance (*p* = 0.088). Similarly, mNDI was not statistically significantly associated with clinical depression when analyzed as a continuous variable (Table 4). No statistically significant differences in the associations were observed in the analyses stratified by sex (Supplement). In addition, the association of mNDI with depressive symptoms nor clinical depression did not differ by BMI. The sensitivity analysis showed results similar to those of the main analysis. When excluding participants taking any psychiatric or mental health medication at baseline (cross-sectional analysis *n* = 32, prospective analysis of elevated depressive symptoms *n* = 8, and of clinical depression *n* = 8), the cross-sectional associations between mNDI and depressive symptoms remained statistically significant and were similar to the primary findings (Model 1, OR 0.970 [95% CI 0.953–0.987], *p* < 0.001; Model 2, OR 0.970 [95% CI 0.953–0.987], *p* < 0.001; Model 3, OR 0.977 [95% CI 0.959–0.994], *p* = 0.010). Similarly, associations at BDI-13 cut-offs 1 and 5 were significant in Model 1 (cut-off 1: OR 0.966 [95% CI 0.938–0.994], *p* = 0.019; cut-off 5: OR 0.931 [95% CI 0.886–0.977], *p* = 0.004) and Model 2 (cut-off 1: OR 0.965 [95% CI 0.937–0.993], *p* = 0.016; cut-off 5: OR 0.943 [95% CI 0.897–0.992], *p* = 0.022), but significance diminished in Model 3 (cut-off 1: OR 1.002 [95% CI 0.974–1.031], *p* = 0.888; cut-off 5: OR 0.979 [95% CI 0.930–1.030], *p* = 0.406). No significant associations were observed at the BDI-13 cut-off of 9 or for clinical depression, consistent with the original results.

## Discussion

We found that higher adherence to a Nordic Diet, assessed with mNDI, was associated with a lower prevalence of depressive symptoms in the Finnish adult population. In addition, higher adherence to Nordic Diet was associated with a lower risk of elevated depressive symptoms over an 11-year follow-up at the lowest BDI‑13 cut‑offs, although these associations were weakened after adjustments for lifestyle factors. There was also some indication of a U-shaped association with the risk of clinical depression diagnosis over the 11-year follow-up, although the p-value for nonlinearity did not reach statistical significance. However, the sample size was small, and the number of cases was limited. In addition, although the inverse association between the mNDIand elevated depressive symptoms appeared somewhat stronger in women, formal tests indicated no statistically significant interaction by sex (Supplement).

The results align with previously published cross-sectional studies conducted in Nordic countries [11, 14]. In a Norwegian study with 5731 participants, a higher a priori diet quality score was linked to a 17% lower risk of case-level depression, as measured by a self-administered questionnaire, in men and a 29% lower risk in women [11]. The most recent study among 2603 middle-aged men from Northern Savo, Finland, found that greater adherence to the Nordic diet, assessed using a healthy Nordic diet score (HNDS), was associated with lower depressive symptom scores [14]. In the Norwegian study, the diet quality score was calculated using FFQ-derived tertiles of six food groups, but it excluded alcohol intake and the fatty acid ratio [11], which are part of the mNDI. The scoring of red meat intake also differed from the mNDI, with the middle tertile receiving the highest score to reflect recommended moderate consumption [11]. In contrast, the scoring of HNDS [15] aligns with that of mNDI, as both are based on BSDS [22]. Similar results have also been reported from studies using *a posteriori* diet analysis [11, 41]. The Norwegian study found that women who reported a healthy dietary pattern, which included vegetables, salads, fruits, rice, pasta, cereals, fish, wine, and unprocessed meats, were 32% less likely to have depressive symptoms [11]. Similar associations were not observed in men [11]. In contrast, a Finnish study involving 1,003 middle-aged men from Northern Savo, Finland, found that a prudent diet, characterized by high intake of vegetables, fruits, whole-grain bread, poultry, berries, low-fat cheese, and fish, was associated with a 25% lower likelihood of elevated depressive symptoms [41]. Araste et al. [13] also reported an inverse association between adherence to the Nordic diet, assessed using the Healthy Nordic Food Index (HNFI) [42], and depressive symptoms in an Iranian case-control study (*n* = 240) among recovered COVID-19 patients. Another Iranian study among 181 healthy women aged 18–25 found no association between HNFI and depressive symptoms [12]. It is noteworthy that the study by Araste et al. [13] was a case-control study, and the association was observed only in the case group (*n* = 120). Also, the studies by Abbaszadeh et al. [12] and Araste et al. [13] were conducted outside the Nordic countries, where habitual dietary patterns differ from those of the Nordic diet. These contextual differences may limit comparability, as the health effects of dietary patterns can depend on regional food cultures, the availability of specific foods, and long‑term dietary traditions [7].

We did not find significant links between mNDI scores and clinical depression. Only one previous study has been published on the association between the Nordic diet and the risk of clinical depression. This prospective study involving 1003 middle-aged or older men from Northern Savo, Finland, reported that a prudent dietary pattern (described above) was associated with a 33% lower risk of clinical depression over 16.5 years of follow-up [41]. Differences in study findings may be partly explained by methodological differences in diet assessment: we employed an a priori diet quality score, which relies on predefined dietary criteria and may not fully capture population-specific combinations of foods relevant to mental health, whereas Ruusunen et al. [41] used an *a posteriori* dietary pattern analysis, which identifies patterns from actual eating behaviors [7]. Furthermore, variations in follow-up periods may have contributed to the inconsistent results. It might take a longer time to observe the cumulative influence of diet on the depression risk.

However, we observed an inverse relationship between mNDI and elevated depressive symptoms over an 11-year follow-up, with cut-offs of 1 and 5 on the BDI-13, suggesting that higher adherence to a Nordic diet might decrease the likelihood of mild depressive symptoms. The lack of evidence of a corresponding association between diet quality and clinical depression may indicate that diet plays a more prominent role in milder forms of depressive symptoms. Although previous studies provide evidence that higher adherence to a Mediterranean-type diet is associated with a lower risk of self-reported depressive symptoms, there is no clear dose–response relationship [9], which may partly explain why we did not find an association between mNDI and clinical depression. Nonetheless, the attenuation observed in the association between mNDI score and elevated depressive symptoms after adjusting for lifestyle factors suggests that these behaviors, such as physical activity, smoking, and BMI, may partly explain the association. This aligns with evidence that lifestyle behaviors tend to cluster, with healthier dietary patterns usually occurring alongside other positive behaviors, while less healthy behaviors tend to group together [43]. Therefore, some of the links between diet quality and depressive symptoms may reflect broader lifestyle patterns rather than diet alone, emphasizing the need to consider co-occurring behaviors in observational studies.

This study has important strengths that enhance the robustness and relevance of its findings. A key strength is the use of a large population‑based dataset, which ensures broad coverage across sexes, socioeconomic backgrounds, and regions. However, selection bias remains a concern; individuals who participate in health and well-being research tend to be healthier, more motivated, or more health-conscious than non-participants [44]. Moreover, since population weights were not applied, the generalizability of the findings to the entire Finnish population may be limited. Nonetheless, the large and representative sample of Finnish adults enables a valuable analysis of these phenomena across the general adult population. The 11-year follow-up period allowed the evaluation of long-term outcomes and temporal associations. Furthermore, the availability of comprehensive data on potential confounding factors enabled adjustment for sociodemographic, socioeconomic, and lifestyle factors. Moreover, diagnostic accuracy regarding depression was strengthened by the use of the diagnostic interview (M-CIDI), a widely recognized diagnostic tool, and, to ensure reliability, all assessments of depressive symptoms were conducted with validated instruments.

Food intake was assessed using a validated FFQ [18, 19]. The use of FFQ is ideal for large population-based studies since it effectively captures long-term dietary patterns cost-effectively and enables ranking individuals by overall diet quality [45]. However, FFQs have well-known limitations. They rely on self-reported data and participants’ recall accuracy of their typical dietary habits, which can introduce recall bias and may lead to measurement errors. Furthermore, there is a potential for misreporting socially desirable or undesirable foods. To reduce measurement error, the instructions for completing the FFQ were provided to participants in person, including predefined portion sizes to help accurately estimate servings. Originally, we analyzed associations using BDI-13 cut-offs of 1, 5, and 9. To elucidate potential misreport of the dietary intake by depressed individuals, we excluded the top either 10% or 20% of participants with a higher BDI-13 score and repeated the analysis. This analysis showed attenuation of the association across all models (for exclusion by 20%, *p* = 0.089, Model 1; *p* = 0.064, Model 2; *p* = 0.086, Model 3, as well as for exclusion by 10%, *p* = 0.098, Model 1; *p* = 0.076, Model 2; *p* = 0.112, Model 3) (Supplementary Table 1). We also performed a sensitivity analysis by excluding participants with extreme daily energy intake, and the results were similar to those from the entire sample (Supplementary Table 2). This indicates that extreme energy intake values did not significantly affect our findings. Although sensitivity analyses partially supported the robustness of the findings, residual confounding cannot be ruled out. While these analyses reduce concerns related to dietary misreporting, unmeasured or imperfectly measured factors may still have contributed to the observed associations.

In addition, a few limitations should be acknowledged. The original dataset included approximately 8000 participants aged 30 years or older, of whom over 1200 did not participate in the health examination. From those participating, nearly 800 did not report their baseline dietary data. In addition, missing baseline data on depressive symptoms and clinical depression diagnosis (*n* = 515) reduced the sample size even further.

A notable limitation of the prospective analysis was the high amount of missing follow-up data on depressive symptoms (*n* = 1163) and clinical depression diagnosis (*n* = 1089), leaving fewer than 2000 participants for the analyses. The most common reasons for discontinuation in the Health 2011 survey were participants’ refusal and inability to contact them [46]. The loss of data due to missing information in this study may have introduced selection bias if participants who discontinued differed systematically in diet quality, depressive symptoms, or clinical depression from those who remained. It is common for individuals with poorer health or more severe symptoms to withdraw from cohort studies [47], potentially leading to an underestimation of true associations. Nonetheless, the conclusions should be interpreted with caution, given the reduced sample size.

Although cross-sectional studies can be affected by the bidirectional relationship between diet and depressive symptoms, our prospective results suggest that better diet quality may be linked to a lower risk of developing mild depressive symptoms. These findings highlight the potential role of diet quality in mental health, though further research with multiple assessments is needed to clarify timing and establish causality. Future longitudinal studies with larger sample sizes could strengthen the evidence connecting diet quality and depression, supporting preventive mental health efforts. Additionally, steps should be taken to increase participation and engagement in these studies. Moreover, research should further explore the mechanisms linking diet and depressive symptoms and evaluate whether combined lifestyle interventions, rather than diet alone, are more effective in reducing the risk of depressive disorders. From a public health perspective, these findings suggest that achievable improvements in moderate-to-high diet quality are beneficial, while also recognizing that diet alone may not be sufficient to alter risk if other factors (e.g., physical activity, smoking, BMI) are considered. The results emphasize the importance of including nutrition in preventive mental health strategies alongside other lifestyle factors.

## Supplementary Information

Below is the link to the electronic supplementary material.

Supplementary Material 1

Supplementary Table 1

Supplementary Table 2

## Data Availability

The data that support the findings of this study are not openly available due to reasons of sensitivity and are available from the corresponding author upon reasonable request. Original data are in controlled access data storage at the Finnish Institute for Health and Welfare.
