# Supplementary material for: Higher adherence to a Nordic diet is associated with reduced risk of mild depressive symptoms: a population-based analysis
Source: Eur J Nutr. 2026 Oct 7;65(8):268. doi: 10.1007/s00394-026-04107-3 (PMC13645869; doi:10.1007/s00394-026-04107-3)
Supplement: Supplementary file 1 [file 394_2026_4107_MOESM1_ESM.pdf]

Higher adherence to a Nordic diet is associated with reduced risk of mild depressive symptoms: a population-based analysis. European Journal of Nutrition.

Mylläri J*, Virtanen JK, Kaartinen N, Jääskeläinen T, Nuutinen O, Absetz P, Männistö S, Ruusunen A, Partonen T.
* Institute of Public Health and Clinical Nutrition, University of Eastern Finland, email: johanna.myllari@uef.fi

Depressive symptoms/clinical depression	Odds Ratio (95% CI)	P-value	P-interaction
**Cross-sectional:**			
All participants [n=4697, 1746 events (37.2%)]	0.976 (0.959 - 0.994)	0.008	
Male [n=2568, 747 events (29.1%)	0.991 (0.965 - 1.018)	0.500	
Female [n=2941, 999 events (33.9%)	0.965 (0.942 - 0.989)	0.004	0.207
**Prospective:**			
**BDI-13 cut-off 1**			
All participants [n=1788, 994 events (55.6%)]	0.972 (0.943 - 1.001)	0.061	
Male [n=889, 474 events (53.3%)]	0.993 (0.951 - 1.038)	0.587	
Female [n=899, 520 events (57.8%)]	0.954 (0.915 - 0.996)	0.031	0.159
**BDI-13 cut-off 5**			
All participants [n=1788, 160 events (8.9%)]	0.962 (0.914 - 1.013)	0.138	
Male [n=889, 67 events (7.5%)]	0.985 (0.911 - 1.066)	0.710	
Female [n=899, 93 events (10.3%)]	0.953 (0.891 - 1.019)	0.160	0.658
**BDI -13 cut-off 9**			
All participants [n=1788, 46 events (2.6%)]	0.991 (0.905 - 1.085)	0.845	
Male [n=889, 21 events (2.4%)]	1.128 (0.983 - 1.128)	0.086	
Female [n=899, 25 events (2.8%)]	0.900 (0.795 - 1.018)	0.095	0.033
**Clinial diagnosis**			
All participants [n=1862, 82 events (4.4%)]	0.995 (0.926 - 1.068)	0.995	
Male [n=932, 28 events (3.0%)]	1.052 (0.927 - 1.194)	0.430	
Female [n=930, 54 events (5.8%)]	0.968 (0.887 - 1.056)	0.462	0.578

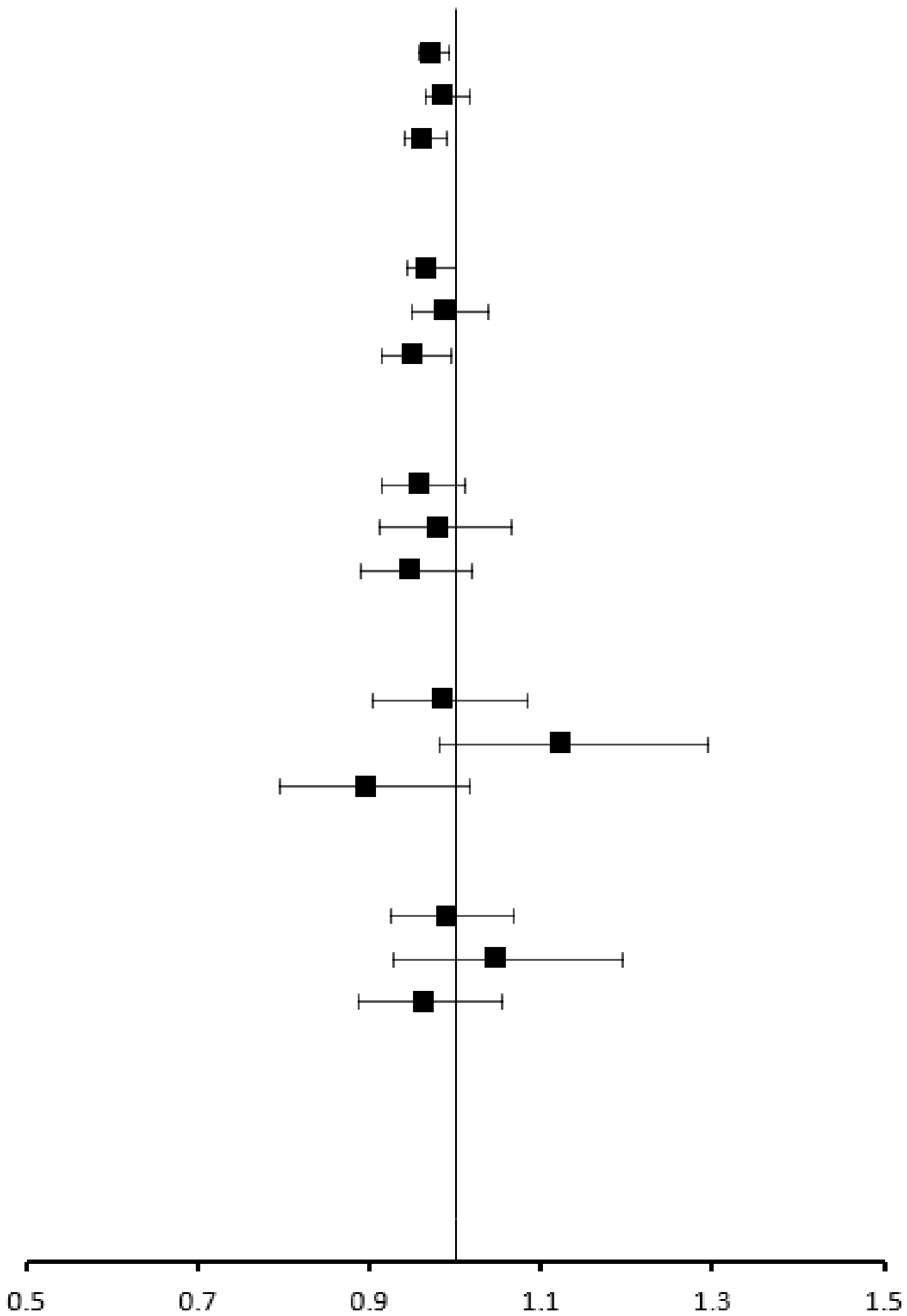

Supplement. Sex-stratified odds per one-point increase in mNDI (year 2000) for depressive symptoms (years 2000, 2011) and clinical depression (year 2011).
P-values for differences in the numbers of events between men and women: Cross-sectional, $p<0.001$; BDI-13 cut-off 1, $p=0.057$; BDI-13 cut-off 5, $p=0.039$; BDI-13 cut-off 9, $p=0.655$; clinical diagnosis, $p<0.003$.
