# Supplementary material for: Higher adherence to a Nordic diet is associated with reduced risk of mild depressive symptoms: a population-based analysis
Source: Eur J Nutr. 2026 Oct 7;65(8):268. doi: 10.1007/s00394-026-04107-3 (PMC13645869; doi:10.1007/s00394-026-04107-3)
Supplement: Supplementary file 2 [file 394_2026_4107_MOESM2_ESM.docx]

| **Supplement Table 1**. Odds ratios for elevated depressive symptoms based on a BDI-13 cut-off score of 1 and mNDI score treated as a continuous variable, while excluding the top 20% and 10% of depressive symptom scores. | | |
| --- | --- | --- |
| **Excluding the top 10%** (N=1628) | | |
| **BDI-13 cut-off 1** | **OR (95% CI)** | **p-value** |
| Model 1 | 0.975 (0.946-1.005) | 0.098 |
| Model 2 | 0.973 (0.944-1.003) | 0.076 |
| Model 3 | 0.975 (0.945-1.006) | 0.112 |
| **Excluding the top 20%** (N=1543) | | |
| **BDI-13 cut-off 1** | **OR (95% CI)** | **p-value** |
| Model 1 | 0.974 (0.944-1.004) | 0.089 |
| Model 2 | 0.971 (0.941-1.002) | 0.064 |
| Model 3 | 0.973 (0.941-1.004) | 0.086 |
| Abbreviations: BDI-13, Beck Depression Inventory-13; mNDI, modified Nordic Diet Index.  Model 1 adjusted for energy intake, age, and sex.  Model 2 adjusted for Model 1 and education level and marital status.  Model 3 adjusted for Model 2 and physical activity level, regular smoking, and BMI. | | |
