# Supplementary material for: Higher adherence to a Nordic diet is associated with reduced risk of mild depressive symptoms: a population-based analysis
Source: Eur J Nutr. 2026 Oct 7;65(8):268. doi: 10.1007/s00394-026-04107-3 (PMC13645869; doi:10.1007/s00394-026-04107-3)
Supplement: Supplementary file 3 [file 394_2026_4107_MOESM3_ESM.docx]

| **Supplement Table 2**. Odds ratios for elevated depressive symptoms based on BDI-13 cut-off scores of 1 and mNDI scores treated as continuous variables, while excluding extreme energy intake values. | | |
| --- | --- | --- |
| **Cross-sectional** (N=4638) | **OR (95% CI)** | **p-value** |
| Model 1 | 0.971 (0.954-0.989) | <0.001 |
| Model 2 | 0.971 (0.953-0.988) | <0.001 |
| Model 3 | 0.977 (0.959-0.995) | 0.008 |
| **BDI-13 cut-off 1** (N=1773) | **OR (95% CI)** | **p-value** |
| Model 1 | 0.964 (0.937-0.993) | 0.015 |
| Model 2 | 0.964 (0.936-0.993) | 0.014 |
| Model 3 | 0.970 (0.941-1.000) | 0.05 |
| **BDI-13 cut-off 5** (N=1773) | **OR (95% CI)** | **p-value** |
| Model 1 | 0.928 (0.884-0.975) | 0.003 |
| Model 2 | 0.941 (0.896-0.989) | 0.017 |
| Model 3 | 0.961 (0.912-1.011) | 0.126 |
| **BDI-13 cut-off 9** (N=1773) | **OR (95% CI)** | **p-value** |
| Model 1 | 0.950 (0.870-1.037) | 0.145 |
| Model 2 | 0.974 (0.892-1.064) | 0.634 |
| Model 3 | 0.984 (0.898-1.078) | 0.773 |
| **Clinical depression** (N=1846) | **OR (95% CI)** | **p-value** |
| Model 1 | 0.984 (0.919-1.053) | 0.634 |
| Model 2 | 0.982 (0.917-1.052) | 0.607 |
| Model 3 | 0.991 (0.923-1.065) | 0.809 |
| Abbreviations: BDI-13, Beck Depression Inventory-13; mNDI, modified Nordic Diet Index.  Model 1 adjusted for energy intake, age, and sex.  Model 2 adjusted for Model 1 and education level and marital status.  Model 3 adjusted for Model 2 and physical activity level, regular smoking, and BMI. | | |
